# Academic resilience in UK pharmacy education – a pilot study applying love and break up letters methodology

**DOI:** 10.1186/s12909-023-04380-4

**Published:** 2023-06-15

**Authors:** Andrew Mawdsley, Sarah C. Willis

**Affiliations:** 1 Division of Pharmacy and Optometry, University of Manchester, Manchester, M13 UK; 2 Management and Policy Division, Alliance Manchester Business School, University of Manchester, Manchester, UK

**Keywords:** Resilience, Academic resilience, Lived experience, Adversity, Burnout, Curriculum, Hidden curriculum, Pharmacy, Pharmacy education, Wellbeing, Mental health

## Abstract

**Introduction:**

Academic resilience is seen as a positive attribute that supports academic attainment and protects against attrition and burnout. Studies have reported that UK pharmacy students have lower academic resilience and wellbeing than the general UK student population but the reasons for this have not been established. This study pilots the use of a novel methodology, love and break-up letter methodology (LBM), to explore these issues focusing on the lived experience of pharmacy students.

**Method:**

Final year undergraduate pharmacy study were purposely recruited to the study. Employing LBM, each participant was invited to write reflective love and break-up letters to their academic resilience in higher education during a focus group. Letters and transcripts of subsequent focus group discussion on the feelings and ideas expressed in the letters were thematically analysed.

**Results:**

Three meta-themes were identified within the data; the curriculum as gas lighting; the curriculum as abusive; and the curriculum as controlling. Students described how the curriculum diminishes academic resilience by working against their sense of agency and self-esteem. A constant threat of failure emerged as defining the student lived experience; students felt controlled by a curriculum with negative impacts on wellbeing and perseverance.

**Discussion:**

This is the first study to use LBM to explore academic resilience in UK pharmacy students. The results provide evidence that some students view the pharmacy curriculum as a source of relentless adversity that is responsible for promoting a hidden negative connection between students and their education. Further study is required to determine if the results can be generalised across the UK pharmacy student body to explain why UK pharmacy students have lower academic resilience than other UK university students and the steps needed to improve academic resilience in UK pharmacy students.

## Introduction

Resilience is a positive predictor of wellbeing [1] and is generally viewed as an underpinning trait in bouncing back from setbacks and thriving in adversity. Resilience is a dynamic set of protective factors [2, 3] reflected in how one responds to difficulty and approaches challenges in the future. In education this would include ongoing attainment after hurdles such as failure or progression challenges [4]. Academic resilience, defined as ‘a capacity to overcome acute and/or chronic adversity that is seen as a major threat to a student’s educational development’ [4] is an attribute to be promoted in learners since it is associated with improved self-confidence, performance, coping and reduced attrition [5, 6]. In this way, resilience protects from burnout, emotional exhaustion [7] and promotes academic success [6, 8].

Research investigating pharmacy student academic resilience has previously taken a quantitative approach [1, 9, 10] using psychometric scales. A study by Cassidy et al. [1] found that United Kingdom (UK) pharmacy students have lower academic resilience than students from other academic disciplines, that poorer resilience is associated with lower wellbeing, and that gender differences in academic resilience exist, with female students more likely to suffer negative emotional consequences of adversity and male students less likely to display help-seeking behaviours. Pharmacy student resilience was also shown as decreasing over time [1, 10]. What has yet to be studied is the lived experience of pharmacy student’s academic resilience using a qualitative, interpretive lens to explore this in more depth.

One novel approach to understanding students’ lived experience is love and break-up letter methodology (LBM), which has recently been used in medical education to study empathy [11]. LBM was originally developed to explore a user’s emotional response and feelings towards interactions with a technology. Through the act of writing a letter, a participant details and expresses their feelings and as such this letter writing is a way in which researchers can start to understand collective experience through the production of reflective narratives [12]. This method is both constructivist and constructionist, as an individual uses reflective writing to articulate their feelings and as a group when meaning is explored and co-constructed in a discussion of the letters written [12]. In this way meaning is constructed of the phenomenon and the reality of it can be understood.

Since resilience is a set of dynamic non-cognitive traits (consisting of self-efficacy, planning, control, composure, persistence), [2] analysis of feelings and emotions about resilience generated through the art of creative and reflective writing where participants outline, explain and make sense of their conceptions of academic resilience (positive and negative) provides a lens through which it is possible to view a participant’s underpinning set of ideas and opinions in relation to their experience of resilience.

Self-determination [13] is a theory in which to explore the manifestation of a learners’ social and emotional competence (SEC) (perceptions and motivations) as a mechanism to understand academic resilience and wellbeing. SEC posits that satisfaction of social-emotional needs (autonomy, social competence, relatedness and a sense of being connected to/cared for by peers and teachers) predicts self-determination. Self-determination in turn is a predictor for a person’s autonomous motivation, adaptive behavioural traits (resilience) and wellbeing [8, 14].

The study reported here used LBM to explore why pharmacy students report poor academic resilience, why it declines over the four years of study on the Master of Pharmacy (MPharm) degree, and how it is linked to well-being. As a study piloting a new method for investigating the student experience, our intention here is also to share details of how a novel approach to data collection can be useful for exploring the student experience.

## Methods

Purposively, students from the final year of a four year MPharm degree (n = 136) at one UK higher education institute were invited to take part in a pilot study using LBM. Recruitment was facilitated through a study advertisement emailed to the year 4 student distribution list. Those who responded to the advertisement were provided with more information about the study and, if interested, were asked to consent to take part.

Data collection for the study took place in a Zoom meeting room®. On arrival in the room, participants were first briefed on the process of LBM and the subject of the letters – academic resilience and what that means in relation to their programme of study – to stimulate thoughts on the topic under investigation. A sample student letter was then read aloud to support participants’ understanding of the method and also to provide an example of how the study was exploring the concept under investigation. Once this was completed, participants were given 30 min to write a love and/or break-up letter to their resilience in relation to their curriculum and their experiences over the MPharm programme. After working individually on their letter(s), participants were brought together in a group, with each participant reading their letters(s) aloud to the group. While participants were reading out their letter(s), the group facilitator made notes about their content, and then used these notes as prompts to seed further questioning in a focus group discussion, stimulating exploration of the themes, feelings and ideas contained within the letters. The whole session was audio recorded and a transcript produced using caption capture within Zoom® which was checked for accuracy against the recorded audio. The letters and the transcript of the focus group acted as the data set and together these were thematically analysed using an iterative comparative process to identify meta-themes [15]. The six steps of thematic analysis were employed; familiarise with the data, generate initial codes, search for themes, review themes, define and name themes and produce the report. The researchers independently analysed the data and then compared codes through reflexive discussion in an iterative process to achieve a consensus in analysis and understanding of the themes.

Ethical approval was sought and obtained from the University of Manchester; project ID: 2021-11327-18272.

## Results

Eight final year students took part in the pilot study (seven identified as female and one identified as male). Most (n = 7) wrote one letter, and following confirmation from participants during the focus group discussion, these have been analysed as a break-up letter. The remaining participant wrote both a love letter and a break-up letter.

What emerged during data analysis, was that both letters and the discussion on academic resilience surfaced negative emotions. Participants described how the MPharm curriculum adversely impacted their academic resilience in multiple ways, and that factors associated with building resilience (confidence, coordination, control, composure and commitment) [2] were also impacted, lowering their self-determination. Experiences of academic resilience reported by participants can be considered in relation to three meta-themes identified in the dataset; (1) The curriculum as gas lighting, (2) The curriculum as controlling and (3) The curriculum as abusive. Each of these themes is considered in more detail next.

### The curriculum as gas lighting

Gas lighting is defined as “psychological manipulation of a person usually over an extended period of time that causes the victim to question the validity of their own thoughts, perception of reality, or memories and typically leads to confusion, loss of confidence and self-esteem, uncertainty of one’s emotional or mental stability, and a dependency on the perpetrator” [16]. Accounts of gas lighting were present in participants’ descriptions of a curriculum which proposes to give students the tools to succeed whilst actively working against them to achieve success, with no consequences for the curriculum if students face adversity. Participants described their treatment at the hands of the curriculum as undermining their self-esteem and sense of self:

“Every year we encounter some difficulties, some made my life so miserable I wanted to just give up. We had our very first argument on the way you write my name … my name is WXYZ, WX is my Chinese name, Y is my English name, Z is my last name. You address me as W, which is the first part of my Chinese name … how can you just pick the first part of my first name and call it a name? When I raised this issue to you, you just dismissed it as you said this is how you work. That was the very first time I felt left behind and unrecognised” (Letter 5, Participant 5)

“You try to teach us to be professionals but I don’t see that in you, every time we have a request or ask for support the answer is “you are full time students, go figure something out”, or “we have personal lives too” or “we don’t get paid to do that”, “you are not first years anymore” (Letter 6, Participant 6)

Where participants indicated that the curriculum had been positive in building resilience this was viewed as bittersweet. Participants felt that they were resilient despite the curriculum working against their self-determination. In this way, the curriculum was experienced as manipulating them into committing to a relationship offering little in return:

“Although there were times that I wish I didn’t commit to this relationship, you have allowed me to grow and become a more resilient person. Nevertheless, there are still so many times I felt unsupported and discouraged and I really hope that you would be able to slow things down and allow myself to have a break without me feeling like a disappointment to you” (Letter 3, Participant 3)

### The curriculum as abusive

Learners’ letters describe themselves as navigating their way through a curriculum that was experienced as stressful. This stress was conceptualised in relation to being under a constant threat of failure. Participants feared failing assessments and the repercussions of failing, which was viewed as not being able to practise as a pharmacist in the future. Learners experienced their position as a learner as precarious, which was damaging to their self-esteem and academic resilience.

“You told me I would not be with you after 4 years if I was to fail, there will be no second chance. You would just leave me behind and all the hard work I have done will be gone and the future I was expecting to have way before I met you will be gone as well. It was just a let-down at that point, but it was not a surprise as you have always been this way. I just hope you can improve your way of handling people’s hardships and learn how to be more forgiving, for the sake of the next person you meet” (Letter 5, Participant 5)

“I have felt confused by your responses to me, often wondering why a degree focussed on the care of others could be so uncaring. My insignificance to you is only magnified by the constant fear of losing you; I have been so dependent on you however, one slight academic blip, and I am no longer needed” (Letter 7, Participant 7)

In the context of the stress associated with failure, students were critical of a lack of concern and emotional support they anticipated they would receive from educators;

#### Participant 4

“I think the worst is when I failed exams in the past, it’s very much like you get the email through they say, um you’ve failed, you need to resit otherwise you’re off this course. They don’t offer you any like emotional support … They don’t take anything else into account they just send you an email saying you failed**…** Why do they send results out on a Friday at 5pm? And then you can’t contact anyone on a Saturday and Sunday. They literally send results, then basically say “sorry if you’ve done really bad, I’m having a good weekend, I won’t reply to you”. If I’ve got my results and I’m crying who am I supposed to contact? There’s no support and you have the whole weekend to stew over it in your head. If you fail something, I wish my advisor would know and send a personal email.”

#### Participant 2

I think what’s the hardest thing about our course is, every single time you sit an exam, you feel so much pressure, because if I fail this, I’ve got one more attempt. And then that one more attempt, it’s like held over your head. It’s like a “you shouldn’t need it, you need to pass first time, I don’t care how you do it, just pass that exam first time, do really well, and then you won’t need it”. Everyone is going to fail at one point, especially on a course that’s hard … They have never taught that to you, never communicate that to you that you are going to fail.”

Participants felt the curriculum perpetuated an idealised version of the student experience, where failure was shameful and failing students were marginalised. This was viewed as a defining principle of curriculum design where the programme was structured to prevent academic progression. This worked to constrain or deny students’ agency;

#### Participant 2

“They make it seem so much like, “you’re the only one that’s failed, like this is you, it’s not us, it’s not our exam. We’ve not made them really hard. It’s you, you’ve not put the effort in”. When 9 out of 10 times, it’s like another 20 people in the year that have failed but you just wouldn’t know that. Yeah, and it’s hard to build that resilience when it feels like you’re the reason it’s failing, it’s all your fault. That’s how it feels when they phrase it to us. “It’s your fault that you’re failing”.

Coping with the threat of failure put a strain on students’ wellbeing, mental health and personal growth; it was described in participants’ letters as the price paid to stay in the relationship (that is, to remain in the programme). Participants urged the curriculum to change so that future relationships would thrive, and provide a context in which resilience was developed;

“Thinking back to 2nd and 3rd year with you, I don’t know how I didn’t do anything impulsive with all the stress there was for me. I am thankful that I didn’t make any bad decisions like harming myself, but I wish you just gave me more time to get to know myself and get settled into this new phase of life a bit better” (Letter 2, Participant 2)

“It made me burnout so hard it took me a year to recover from it. It also made me mentally ill and it broke my heart when I realised you didn’t care enough or as much as I did. Only if you knew I rejected [competitor University] for you” (Letter 6, Participant 6)

“As our time together draws to a close, I am left with a bittersweet taste in my mouth; you have taught me enough to be able to navigate a successful pharmaceutical career, for which I will always be grateful. However, I urge you to learn from the frustrations and disappointments you have caused me along the way, so you can produce professionals confident in not just their abilities, but also in themselves” (Letter 7, Participant 7)

Participants’ letters imagined a future in which they had moved on from the abusive relationship they experienced as a pharmacy student;

“Now I am in my 4th year with you and I am more than ready to leave and start a new beginning of my life. You have been too difficult. The years with you have been years of growth, but pain and regrets. I sometimes wish there was somebody to help me out with the relationship I had with you, but maybe I had to go through everything to learn” (Letter 2, Participant 2)

“I have changed a great amount whilst in your company, and whilst I may now be prepared to become a competent healthcare professional, this process has come at a price ... You have often been cold and unfeeling, which coupled with high expectations, has led to a diminished sense of self confidence. I have often felt like just another student to you, rather than another person, I wonder if you even know my name?” (Letter 7, Participant 7)

While the curriculum was viewed as abusive, participants were able to act with some agency within their peer networks. Thus they considered peer networks as a place where they could mobilise more active strategies to protect against and cope with adversity and build their resilience;

**“**I also think it’s massively from like support from friends that I didn’t think I could have got through the four years without friends, without any support from Uni [university], like I don’t think Uni [university] understand that how much of our year probably struggle or how much they do they’d never offer support they never offer you things. The people that you turn to the most I’d say is my friends on the course” (Letter 2, Participant 2)

While offering a space within which to build resilience as a more active agent, because of experiences of gas lighting, students rarely discussed academic failure within their peer support networks. As a consequence, the lived experience of the curriculum was isolating;

#### Participant 2

“I think as well, no one talks about it. No one talks to each other being like, “oh I failed that”, no one wants to admit that. Like I’ve failed something every year, I’ll be the first to admit it, like, yeah, I shouldn’t be here as well. But no one talks to each other and says, “I failed that”. Uni [university] doesn’t create an environment where students feel, like open about failure with each other. But we are probably the biggest support we can give to one another, because most of us have failed at least one thing, at every single point but they don’t like encourage people to talk before resits to get support from each other. They don’t encourage study groups if you’ve got a resit. There’s never the “it’s okay to fail”. And it is okay to fail”.

Relationships with staff were occasionally seen as a useful source of support and context within which to build resilience, although these relationships were often underdeveloped and transactional;

#### Participant 1

“I think one thing that I like about uni [university], is my academic advisor, but I don’t think I spend enough time with him to get like a good relationship with him. But like when I have a problem, I would go to him, and that would be like one thing that really helped me when I was struggling with things, I would talk to him”.

### The curriculum as controlling

The controlling nature of the curriculum meant that it was experienced as a constraining structure or ‘total world’. Participants described their programme as taking up so much space that they have little agency to develop outside of their studies:

“My flatmates who were studying other degrees were living their normal life, like a normal person. I wish you knew I had life outside this” (Letter 2, Participant 2)

“As I get to know you better, I realised that our life goals were so different, you are demanding yet I wanted to focus more on myself and my self-development. I wanted to have a life outside of you as well” (Letter 3, Participant 3)

Student letters frame the curriculum as a narcissist where self-interest is paramount and their own wants and needs are diminished. Thus, tough love was the defining feature of their relationship;

“As a course – you really suck. You are meant to help me figure out how to balance my work and life and not overtake it. You are obsessed with your prestigious-ness and how famous you are, you have forgotten to take care of me. All you care is the result, your rankings, and the money” (Letter 2, Participant 2)

Participants expressed the lived experience of their studies as one in which their promises and expectations were unfulfilled – and thus the relationship was not what they thought it would be;

#### Participant 4

Coming in and it all being like the [pharmaceutics]. It wasn’t what I expected from a pharmacy course. A lot of us, I think did it, because we want to help people. And the [science] to me was just so far away from what I’d ever expected it to be. And it did feel like sometimes they were saying to you, that if you weren’t a good chemist, you were never going to be a good pharmacist.

## Discussion

This is the first study in pharmacy education to pilot the use of LBM, and whilst not intended to be generalisable or representative, our findings provide qualitative insights into possible reasons why pharmacy students have previously reported poor resilience and wellbeing [1]. It is of note that amongst those taking part in our study, predictors of academic resilience as defined by Martin and Marsh [2] (self-efficacy, control, planning, low-anxiety and persistence) were absent in both the letters and in the focus group discussion, which may explain why outcomes of academic resilience, such as enjoyment of school, participation and self-esteem were also absent [2]. From the viewpoint of self-determination theory, this could be interpreted as pharmacy students lacking social-emotional autonomy, and as a consequence are questioning their social competence, and express poor feelings of relatedness to peers and teachers, which is then related to their low academic resilience and wellbeing. That academic resilience is an artefact of the pharmacy curriculum, and the interaction and experience learners have with it is of concern. This argument is further supported in the actions of seven of the participants who chose to only write a break-up letter to the pharmacy programme, suggesting that their feelings about their experiences of the curriculum are negative in terms of participants’ academic resilience and understanding of it.

Learners express a sense of abuse where the curriculum is in control whilst operating a threat of failure. The overarching reasoning for poor academic resilience is related to assessment methods; the assessment methods operating in this study are cultivating a climate of fear [17]. Competency based assessments which are experiential and authentic to pharmacy practice are considered to ensure competence whilst increasing student confidence [18–20]. Ability-based assessments adopted as a continual student-centred process are shown to support students in their academic development, foster self-determination [21] and are resilience building. There is evidence that employing multiple assessment opportunities as opposed to high-stakes terminal assessments through an assessment for learning ethos, such as using entrustable professional activities (EPAs), applied through continuous experiential learning placements could help foster resilience building and student affinity in learning [22–24]. In the UK, the revised initial education and training standards [25] and the pharmacy education reform programme [26] adopt competency based frameworks and recommend the use of EPAs which may have a positive impact on the themes identified in this study, namely that assessment damages a learner’s sense of accomplishment, confidence and determination and over time diminishes resilience and wellbeing. It is known that UK pharmacy student resilience decreases over time and is intrinsically linked to wellbeing [1] and so adopting performance or ability-based assessments and moving away from high-stakes terminal assessments and increasing experiential learning could have a positive impact on academic resilience and wellbeing.

Applying LBM has provided us with a lens through which to gain sight of the experienced, hidden curriculum in which academic resilience is expected by the curriculum but experienced as a persistent threat of failure rather than being nurtured so that students have the tools to cope with adversity; it is in this sense that learners are not supported to develop those protective factors that would enable them to thrive. As the threat of failure is the overwhelming lived experience in the student psyche and affecting their behaviour students describe how they were at all times operating in a manner to avoid adversity, avoid failure and so not building the psychological tools to respond to the adversity they are experiencing. Because of lack of transparency in attainment and lack of support mechanisms (poor staff-student and student-student relationships) pharmacy education is a lonely place for students to thrive where resilience is expected but not developed.

The underpinning sentiment from the findings is that the curriculum works against the learner’s academic resilience and in doing so the hidden curriculum is at best bittersweet and at worst detrimental to students’ mental health and wellbeing. The main reasons for this stem from the role of assessment, felt by learners as punitive and ruthless; requiring peak performance at all times with no buffer or leeway. Academic resilience is undermined by the curriculum and in particular the assessment in a sense as being against the learner, unsupportive and adversarial with no tolerance for slips or setbacks exposing a damaging assessment strategy and one that does not support learning but rewards achievement. The participants in this study clearly expressed aspects of burnout in relation to this. This finding is in contrast to a recent study by Hanna et al. (2022) [10] which found that high stakes assessments with a high pass mark was resilience building. The difference in findings between this and our own study is that underpinning the curriculum Hanna investigated was the use of formative assessment and simulation (the opportunity to practice in a safe environment), which students viewed as resilience building.

Limitations of this study affect transferability of the findings and include the small number of participants and that data were collected at one site and so the meaning of academic resilience as interpreted by these participants is situated in this one shared experience. LBM is a method that is potentially uncomfortable to participants and so recruitment to the study was difficult and limited the data and potential saturation of themes. The higher number of female participants may be in part due to the method and also means the findings are female-centric but also the demographics of UK pharmacy students; the undergraduate pharmacy student population of the UK is largely female, with less than a third male [27]. It is known that male and females differ in their academic resilience traits and behaviours; that female students attach more negative emotion to adversity [1]. This study supports this finding but given low male representation in the study the findings may not extend to explain the emotions and feelings underpinning the academic resilience of male students who have been found to be less likely to seek help in adversity but also less likely to harbour negative emotions towards academic adversity and setbacks [1].

Participants in this study welcomed the cathartic process of reflection LBM allows and expressed that letter writing, reflecting, narrating, listening and discussing within a group of peers had a positive impact on sense of wellbeing, social connection and in itself is a useful method of honest and realistic student-led dialogue which may be useful for pharmacy educators to consider when designing interventions for supporting student wellbeing and academic reesilience [12]. In this way, the methodology can act as a form of resilience intervention and clearly has a place in future work investigating the hidden curriculum.

## Conclusion

This study presents insights into the psyche of the pharmacy student body, their wellbeing and academic resilience and contributes to our understanding of likely factors creating poor academic resilience amongst pharmacy students reported by others [1, 9]. Educators should focus on: supporting students in developing their self-determination; providing learning targeted at managing their workload; and crucially by reframing assessment as supporting authentic practice-based learning, not as a device to punish them. By giving learners the space and platform to reflect in peer networks in open ways about their wellbeing and to rebalance the terms of the relationship between educators and learners it will be possible to encourage more love and less break up.

## Data Availability

The datasets generated during and/or analysed during the current study are available from the corresponding author on reasonable request.
